# Phonological decoding and orthographic learning in poor and typical adult readers

**DOI:** 10.21203/rs.3.rs-8491793/v1

**Published:** 2026-01-21

**Authors:** Anna Chrabaszcz, Kailee Lear, Corrine Durisko, Julie Fiez

**Affiliations:** University of Pittsburgh; University of Pittsburgh; University of Pittsburgh; University of Pittsburgh

## Abstract

This study investigated whether adults with long-standing reading difficulties (“poor readers”) can acquire new literacy skills in both familiar (English pseudowords) and novel (artificial orthography, AO) systems, and how phonological decoding deficits relate to orthographic learning. Poor readers (n = 17) and matched typical readers (n = 17) completed a decoding task and three orthographic learning tasks (spelling, identification, and lexical decision) in both orthographies. Results showed that poor readers achieved near-ceiling accuracy in learning novel grapheme–phoneme correspondences, yet remained less fluent than typical readers across both orthographies, even after repeated practice. Despite this persistent fluency gap, poor readers performed comparably to typical readers on measures of orthographic learning, indicating that decoding efficiency does not directly constrain orthographic acquisition under conditions of equal exposure. Exploratory cluster analyses revealed heterogeneous profiles among poor readers, with distinct constellations of cognitive strengths and weaknesses shaping decoding outcomes. These findings highlight fluency as the central bottleneck in adult literacy while demonstrating that adult readers with long-standing reading difficulties can form accurate orthographic representations through structured practice. The results also highlight the importance of considering individual variability in both research and adult literacy interventions.

## Introduction

For most literate adults, reading feels so natural that it is often taken for granted. Yet this apparent ease belies the extraordinary complexity of the process, which requires seamless coordination of multiple cognitive and linguistic systems (Castles & Nation, 2006; Vellutino et al., 2004; Wolf et al., 2024). When any of these systems is compromised, reading can break down, resulting in persistent difficulties for a substantial portion of the population—estimates suggest that up to 20% of individuals experience problems with reading (Calhoon, Scarborough, & Miller, 2013; Shaywitz, Shaywitz, & Shaywitz, 2021). Such difficulties rarely disappear with age; rather, many struggling readers continue to lag behind their peers in fluency, comprehension, and vocabulary growth throughout adulthood (Bruck, 1993; Reis et al., 2020; Scarborough, 1984; Swanson & Hsieh, 2009; Undheim, 2009; Vellutino et al., 2004). Meanwhile, the demands of reading often increase across the lifespan, as adult readers encounter new vocabulary in academic, professional, and everyday contexts. Success in meeting these demands carries profound consequences for educational attainment, employment opportunities, and quality of life (de Baldini Rocha & Ponczek, 2011; McLaughlin, Speirs, & Shenassa, 2014; Ritchie & Bates, 2013). Yet because most theories of reading development are grounded in studies of children, we lack a clear understanding of how adults acquire new orthographic skills and how this process is different for individuals with persistent reading difficulties. The present study addresses this gap by examining how struggling adult readers acquire new orthographic knowledge, specifically focusing on their phonological decoding skills and orthographic learning outcomes for known and novel orthographies, and identifying points of potential breakdown.

At the very heart of reading is the ability to extract meaning from print. In alphabetic languages such as English, this process begins with the acquisition of the alphabetic principle—the understanding that visual symbols of the writing system (graphemes) map systematically onto sounds of the spoken system (phonemes) (Byrne & Fielding-Barnsley, 1989; Castles, Rastle, & Nation, 2018; Ehri, 1987). According to the self-teaching hypothesis (Share, 1995; 2008), mastery of the alphabetic principle enables readers to decode novel visual (orthographic) forms into spoken (phonological) forms, thereby leveraging preexisting spoken language knowledge to access meaning. Every successful attempt at decoding an unfamiliar written word strengthens the connections between its orthographic, phonological, and lexical representations, gradually integrating it into the reader’s orthographic lexicon for more efficient recognition. Key to the self-teaching hypothesis is the idea that phonological decoding can happen in an item-based fashion to support learning of new words regardless of the reader’s proficiency, and is, therefore, not only characteristic of child reading acquisition, but also remains important in adulthood because adults continue to expand their vocabulary throughout life (Leach & Samuel, 2007; Nation & Kyongho, 1995). Indeed, phonological decoding skills continue to account for a large amount of variance in reading comprehension and broader literacy outcomes in adulthood (Braze et al., 2007; Talwar et al., 2021; Tighe & Schatschneider, 2016). Moreover, decoding skills reliably distinguish skilled from struggling adult readers: adults with persistent reading difficulties, including dyslexia, consistently perform worse than their peers on tasks such as pseudoword and irregular word reading (e.g., Elbro, Nielsen, & Petersen, 1994; Kitz & Tarver, 1989; Reis et al., 2020), and the performance gap may even be wider in adulthood than in childhood (Pennington et al., 1987). Taken together, these findings highlight phonological decoding deficits as a central cause of reading difficulties that persist into adulthood.

While phonological decoding provides a gateway to reading new words, this strategy is inherently slow and effortful, and cannot by itself support the fluent, automatic word recognition characteristic of adult reading. A typical adult reader can read up to 300 words per minute (Brysbaert, 2019)— a speed far exceeding what is possible through serial, laborious, letter-by-letter decoding. This discrepancy suggests the need for additional mechanisms that enable efficient, fluent reading. One such mechanism is accessing word meaning directly via orthographic representations bypassing the phonological route (Castles & Nation, 2006; Ehri, 2014). Orthographic representations, or memory traces of word-specific grapheme sequences that can be rapidly accessed during reading and spelling (Apel, Henbest & Masterson, 2019; Ehri, 2014), are the result of orthographic learning throughout repeated reading exposure and practice. Robust, well-specified, fully developed orthographic representations facilitate fluent and effortless reading and mark the transition from the system that relies more heavily on phonological decoding to one that relies more heavily on automatic orthographic recognition, especially for frequent, familiar words (Castles & Nation, 2006; Castles, Rastle, & Nation, 2018; Wang et al., 2014). Indeed, orthographic processing skills have been shown to explain unique variance in reading outcomes above and beyond phonological decoding ability (e.g., Badian, 2005; Cunningham, Perry, & Stanovich, 2001; Stanovich & West, 1989).

Whereas skilled readers, both children (e.g., Bowey & Muller, 2005; Share, 2008) and adults (e.g., Chalmers & Burt, 2008; AUTHORS; Ginestet et al., 2020; Maloney et al., 2009), can establish durable orthographic representations after only a few exposures, poor readers struggle to do so even with repeated practice (Bailey et al., 2004; Ehri & Saltmarsh, 1995; Suárez-Coalla et al., 2014). Difficulties may arise from deficits in the initial word decoding during exposure or from weaknesses in the subsequent encoding, storage, or retrieval of orthographic information, which prevents the development of automaticity and, ultimately, affects reading fluency and comprehension. Notably, deficits in the orthographic domain can also occur in readers with relatively intact decoding skills, as in cases of “surface dyslexia” (Bailey et al., 2004; Castles & Coltheart, 1996; Hanley, Hastie, & Kay, 1992). Such individuals perform well on pseudoword reading tasks measuring phonological decoding skills but perform more poorly on tasks requiring retrieval of word-specific orthographic information from memory or connecting it with meaning in the lexicon. For example, they often regularize irregular words (e.g., read “break” as “breek”), and make incorrect choices in homophone judgment tasks (e.g., “Which of these is a vegetable, bean or been?”).

Cumulatively, these findings suggest that reading difficulties can emerge from at least two loci: weaknesses in phonological decoding, which impair accurate word recognition, and weaknesses in orthographic learning, which hinder the formation of stable orthographic representations. Yet these two domains are deeply interconnected: phonological decoding provides access to novel word forms, while orthographic learning consolidates them into the mental lexicon (Share, 1995, 2008). Indeed, research with children shows a strong correlation between decoding accuracy and orthographic learning outcomes (Cunningham, 2006; Cunningham et al., 2002; Share, 2008), and children with dyslexia demonstrate that weaker decoding skills constrain orthographic learning (Share & Shalev, 2004). However, despite clear evidence linking phonological decoding and orthographic learning in children, little is known about how these processes interact in adults with persistent reading difficulties. The present study takes a critical step toward addressing this gap by examining how phonological decoding and orthographic learning operate in adults with and without reading difficulties.

### The Present Study

Orthographic learning is not limited to childhood but continues across the lifespan, as adults regularly encounter and acquire new words incidentally through natural reading experiences. However, the contexts and mechanisms of orthographic learning differ markedly between children and adults. When children encounter a new print word, they usually already know its spoken form and meaning. Their task is therefore to link a novel orthographic form to an existing spoken word (novel orthographic form ◊ known phonological form). Because their knowledge of the writing system is still developing, children rely heavily on their spoken vocabulary and phonological representations to support visual word recognition. Adults, in contrast, often face the opposite situation: they encounter written words whose meaning and pronunciation are unfamiliar (e.g., technical terms, foreign place names, medical prescriptions). In these cases, adults must learn both orthographic and phonological forms simultaneously (novel orthographic form ◊ novel phonological form). At the same time, adults have an advantage of extensive print experience, which provides sensitivity to general properties of the orthographic system, such as letter-position frequencies, sequential dependencies, and orthographic constraints (e.g., the intuition that “febb” is a more likely word in English than “ffeb”) (Apel, Henbest & Masterson, 2019; Castles & Nation, 2006). Such knowledge can facilitate phonological decoding, visual word recognition, and orthographic learning. Yet it remains unclear whether adults with reading difficulties can capitalize on this knowledge, given evidence that dyslexic readers show reduced sensitivity to orthographic regularities (Pitchford, Ledgeway, & Masterson, 2009).

Considering these different scenarios, we implemented two research protocols to examine the different mechanisms of orthographic learning in adulthood: the pseudoword protocol and the artificial orthography (AO) protocol.

The pseudoword protocol uses participants’ native language orthography (English) and emulates a scenario of how adults may encounter new words in real life. In this paradigm, we use the same methodology previously used in studies of orthographic learning in children (de Jong & Share, 2007; Kyte & Johnson, 2006) and adults (Burt & Blackwell, 2008; AUTHORS). In these studies, participants usually learn orthographically legal pseudowords (e.g., “bleaz”, “nurch”) and are later tested on their ability to discriminate target spellings from plausible alternatives (e.g., “bleaz” vs. “bleez”, “nurch” vs. “nerch”) or actively recall them in spelling tasks. Successful recognition or recall indicates that stable orthographic representations have been established. Because pseudowords are constructed in participants’ native language, this approach allows learners to leverage accumulated orthographic knowledge of their native writing system. Importantly, however, reliance on familiar orthography introduces interpretive challenges. For example, if an individual does well on the tests of known orthography, this does not necessarily imply that they will do well on learning new orthographies, like in second language acquisition contexts. Moreover, with struggling readers, this approach may obscure underlying difficulties by reflecting the outcome of years of compensatory strategies, interventions, and reading experiences rather than revealing initial reading deficits.

To address these limitations, researchers have increasingly turned to dynamic training approaches, which assess learning potential rather than accumulated experience (Dixon et al., 2023; Wood, Biggs, & Molnar, 2024). This method has demonstrated high accuracy in identifying individuals with or at risk for reading disorders (Dixon et al., 2023). In dynamic paradigms, participants acquire novel grapheme–phoneme correspondences in an unfamiliar orthography or an artificial orthography (AO) (e.g., that a circle corresponds to sound /b/, a triangle corresponds to sound /i/, etc.) and apply them to decode unfamiliar orthographic forms. This paradigm mimics child reading acquisition, in which learners map new orthographic forms onto known spoken forms (novel orthographic form ◊ known phonological form). Because the phonological forms are already part of the lexicon, participants can draw on their lexical knowledge to drive word recognition. The advantage of such approach is that it highlights latent learning potential rather than end-state performance while reducing confounds due to heterogeneous reading profiles, such as differences in socioeconomic background, literacy opportunities, or prior interventions (Peña, Iglesias, & Lidz, 2001). Dynamic AO paradigms have been successfully applied to assess phonological decoding in children (Aravena et al., 2016; Cho et al., 2020; Horbach et al., 2018) and orthographic learning in adults (Rastle et al., 2021; AUTHORS), but they have not yet been used with adults who struggle with reading. It therefore remains unknown how persistent reading difficulties affect the acquisition of novel grapheme–phoneme associations and orthographic learning in an unfamiliar script.

By combining these two approaches—the English pseudoword protocol and the AO word protocol—we aim to capture distinct but complementary scenarios of orthographic learning in adulthood. The pseudoword paradigm reflects the everyday challenge of acquiring both orthographic and phonological forms for novel words in a familiar writing system. The AO paradigm, in contrast, assesses the ability to acquire novel grapheme–phoneme associations dynamically, while minimizing the influence of prior literacy experience. Together, these methods allow us to address the overarching question of whether individuals with long-standing reading difficulties can successfully acquire new literacy skills, such as learning an unfamiliar orthography. Specifically, we asked:

1. Do persistent reading difficulties impair phonological decoding across both familiar and novel orthographies?
2. Do these difficulties constrain orthographic learning outcomes in familiar versus novel orthographies?
3. How do phonological decoding skills relate to orthographic learning performance across familiar and novel orthographies?

## Methods

### Participants

The study sample consisted of 34 young adults (*M* age = 26.8 years, *SD* = 6.17), including 17 typical readers and 17 poor readers. All participants were right-handed, monolingual native speakers of English. All participants reported no history of psychiatric or neurological conditions (including ADHD or autism). Typical readers were matched to poor readers as closely as possible on age, gender, and education level (see Table 1).

Group assignment was determined through a screening assessment. Participants were classified as poor readers if they met the following criteria: 1) a self-reported lifelong history of reading difficulties, 2) a score greater than 40% on the Adult Reading History Questionnaire (ARHQ; Lefly & Pennington, 2000), 3) substandard performance (standard score < 90) on at least two of four standardized reading subtests of timed and untimed word and pseudoword reading: the Sight Word Efficiency and the Phonemic Decoding Efficiency subtests of the Test of Word Reading Efficiency battery (TOWRE; Torgesen et al., 2012), and the Word Identification and the Word Attack subtests of the Woodcock Reading Mastery Test (WRMT; Woodcock, 2011). If participants had a formal diagnosis of dyslexia (5 out of 17 poor reader participants), they still had to meet all the above criteria to be included in the study.

Participants in the typical reader group were required to score 90 or above on all four subtests listed above, score below 40% on the ARHQ, and report no history of reading difficulties or dyslexia.

In addition to the screening measures, all participants except one completed a standardized test battery that assessed phonological processing (phonological decoding, memory, and rapid automatized naming) (Comprehensive Test of Phonological Processing; CTOPP, Wagner et al., 2013), reading comprehension (Nelson-Denny Reading Test; NDRT, Brown et al., 1993), verbal and nonverbal subtests of the Wechsler Adult Intelligence Scale (WAIS; Wechsler, 1997), and the Color-Word Interference (Stroop) test and the verbal fluency test of the Delis-Kaplan Executive Function System (DKEFS) battery (Delis, Kaplan, & Kramer, 2001). All participants met the general inclusion criterion of scoring 85 or higher on the WAIS Perceptual Organization Index, a measure of nonverbal IQ. Table 1 provides a comparison of group scores across screening and assessment measures.

All participants signed informed consent and were compensated for their participation. The study procedures adhered to the ethical guidelines outlined in the Declaration of Helsinki and were approved by the Institutional Review Board at the University of Pittsburgh (STUDY19070420).

### Experimental protocols

Participants completed two protocols comparing phonological decoding and orthographic learning: an English pseudoword protocol and an AO word protocol, administered approximately one week apart. In each protocol, participants first completed a reading-aloud decoding task to assess phonological decoding. On the following day, they completed three orthographic learning tasks—spelling, identification, and lexical decision. Task design was held constant across protocols to allow direct comparison, with two exceptions. First, the AO protocol included an additional training phase to support learning of the novel script. Second, AO tasks were conducted in-lab while English pseudoword tasks were administered remotely via Zoom and Pavlovia (https://pavlovia.org/).

### Materials

#### English pseudoword protocol.

##### Decoding stimuli.

Sixteen monosyllabic (4–5 letters in length) pseudowords (e.g., “bleaz,” “nurch”) were selected from a previously validated corpus (AUTHORS, Experiment 2) for use in the decoding task. A complete list of the English pseudoword stimuli is provided in Appendix Table A1.

##### Orthographic learning stimuli.

To test participants’ orthographic memory for the encountered pseudowords, each pseudoword was paired with a homophonous alternative that differed only in the vowel grapheme(s) (e.g., “bleaz” vs. “bleez”; “nurch” vs. “nerch”). For the lexical decision task, we added 32 distractors (“no” responses): 16 unpronounceable English letter strings and 16 unknown letter strings in Cyrillic (see Appendix Table A1). Audio recordings of the 16 target pseudowords were produced by a native speaker of American English for use in the spelling task.

#### Artificial orthography (AO) protocol.

##### Artificial alphabet.

Participants learned an artificial alphabet composed of 24 symbols visually resembling ancient runes. Of these, 20 symbols represented 10 English consonant phonemes (/b/, /d/, /k/, /l/, /m/, /n/, /p/, /r/, /s/, /t/), with each phoneme assigned two distinct graphemic realizations. This is analogous to English, where a single phoneme can be represented by multiple graphemes (e.g., /k/ in “Cat” and “Kat”). The remaining four symbols represented six English vowel phonemes (/☒/, /☒/, /æ/, /a☒/, /i/, /e☒/). Specifically, /☒/ and /☒/ were each represented by two distinct symbols; /æ/ and /a☒/ shared a single symbol, and /i/ and /e☒/ shared another. This setup mirrored English orthographic ambiguity, where a single letter can correspond to multiple vowel sounds (e.g., letter ‘a’ in “mat” vs. “mate”). These manipulations allowed for the creation of homophonous word pairs to assess orthographic learning. The full artificial alphabet is provided in Appendix Table A2.

##### Decoding stimuli.

Sixteen English words were selected to be transliterated into the artificial alphabet for the decoding task. They were all high frequency words (*M* = 66.4 ipm, *Range* = 13–228 ipms), four phonemes long, and consisted only of the phonemes used in the AO system (Appendix Table A2). The words were carefully counterbalanced, with each of the four vowel letters appearing in four words. Additionally, 160 words of varying length were selected for training to read in the AO over ten days prior to the completion of the decoding task.

##### Orthographic learning stimuli.

To assess orthographic learning, we created 16 homophonous counterparts for the decoding words by replacing all consonant graphemes with their alternate forms while keeping pronunciation constant. For the lexical decision task, we added two types of distractors (“no” responses): 16 unpronounceable AO letter strings and 16 strings composed of unknown, rune-like symbols (see Appendix Table A2). Pronunciations of AO stimuli were synthesized using the Google Text-to-Speech (U.S. English) Python library.

### Procedure

The study spanned several weeks and combined in-lab visits, Zoom sessions, and asynchronous at-home training. The procedure began with an initial Zoom session during which participants completed screening and cognitive assessments. In a second Zoom session, participants were introduced to the AO system and learned grapheme–phoneme correspondences through guided letter–sound matching and word-reading practice. Training continued until participants reached ≥ 80% accuracy on grapheme–phoneme identification. Participants then completed nine additional days of self-paced online AO reading practice using the same materials, with all sessions audio- or video-recorded to monitor compliance. Following training, participants completed two consecutive in-lab sessions: an AO decoding task on Day 1 and three orthographic learning tasks (spelling, identification, and lexical decision) on Day 2.

About a week later, participants completed the English pseudoword protocol that mirrored the AO procedure. The pseudoword decoding task was administered online via Pavlovia (https://pavlovia.org/) and recorded for compliance and scoring, followed by spelling, identification, and lexical decision tasks administered over Zoom on the subsequent day.

All tasks, whether administered in the lab or online, were programmed in PsychoPy (Peirce et al., 2019).

#### Decoding task.

**Figure 1:**
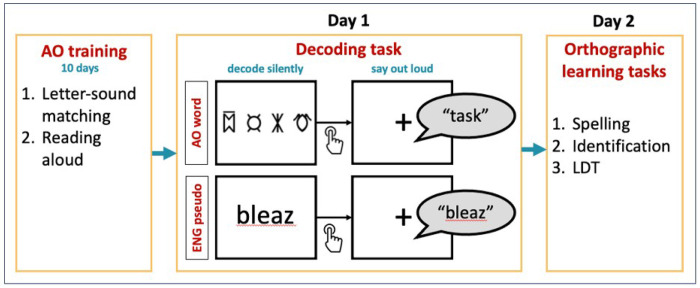
A schematic representation of study procedures and tasks.

In both the AO word and English pseudoword decoding tasks, participants read aloud 16 stimuli (Appendix Tables A1 and A2). Each trial began with the presentation of a stimulus on the screen. Participants were instructed to silently decode the stimulus first, then press a response key as soon as they were ready to produce their response. Upon keypress, a fixation cross appeared for two seconds, during which the spoken response was recorded, thereby separating reading and articulation phases in each trial (Fig. 1). Each stimulus was presented once per block across 16 blocks, yielding 256 trials per participant.

#### Orthographic learning tasks.

To assess orthographic learning of items encountered in the decoding task, participants completed three tasks: spelling, identification, and lexical decision (Fig. 1).

##### Spelling task.

Participants heard an audio recording of a target stimulus (AO word or English pseudoword) and reproduced its spelling. English pseudowords were typed using a standard keyboard, whereas AO words were spelled by selecting symbols from the artificial alphabet displayed on the screen.

##### Identification task.

Participants were presented target words and their homophonous counterparts one at a time. They read them aloud and indicated whether each specific word form had appeared in the decoding task by pressing “yes” or “no.”

##### Lexical decision task (LDT).

Participants judged a mix of pronounceable and unpronounceable letter strings, responding “yes” for pronounceable items (targets and homophones) and “no” for unpronounceable strings. Unpronounceable stimuli served as distractors and were excluded from analyses.

### Analysis

All data analyses were conducted using R (version 4.4.1; R Core Team, 2024). The *lme4* package (Bates et al., 2015) was used to model linear mixed effects (LME) (lmer function) for continuous data, and logistic generalized linear mixed effects (GLME) (glmer function) for binary data (with the “bobyqa” optimizer). Following the recommendations of Barr et al. (2013), we initially specified maximal random-effects structures, including random intercepts and slopes for group by subject and item. If these models failed to converge, we simplified them to include only random intercepts for subjects and items, which accounts for the repeated measures design and participant/item-level variability.

Model output and effect sizes (marginal and conditional R^2^) were generated using the *sjPlot* package (Lüdecke, 2022). Marginal R^2^ reflects the variance explained by fixed effects, while conditional R^2^ reflects the variance explained by both fixed and random effects. Between-group and post hoc comparisons were conducted using Welch’s t-tests to with unequal variances.

Latency data (reading times and lexical decision times) were trimmed within each participant and condition using a 2.5 median absolute deviation (MAD) threshold (Leys et al., 2013) and then log-transformed for statistical analysis. All data visualizations were created using raw (untransformed) values in seconds.

Spelling performance was evaluated using Levenshtein distance, which quantifies the number of insertions, deletions, and substitutions needed to convert a participant’s spelling into the correct target form (with a score of 0 indicating an exact match).

Identification accuracy was assessed using d prime scores, which reflect participants’ ability to discriminate between target forms and homophonous distractors. For each participant, hits (correct identifications of targets), correct rejections (correct rejections of homophones), misses (targets identified as homophones), and false alarms (homophones identified as targets) were used to compute the d prime score.

Exploratory cluster analysis was performed with the *factoextra* package (Kassambara & Mundt, 2020) in R, using enhanced hierarchical clustering and no pre-defined clusters.

All data visualizations were produced using the g*gplot2* and *tidyverse* packages (Wickham, 2016; Wickham et al., 2019).

## Results

### AO learning outcomes

Following ten days of training to read in the AO, both groups of participants successfully learned the artificial alphabet and were able to read AO words with high accuracy: on the final day of training (Day 10), reading accuracy was at ceiling for both groups (typical readers: *M* = 0.95, *SD* = 0.08; poor readers: *M* = 0.95, *SD* = 0.06). Post-training letter-sound matching performance was also at ceiling (typical readers: *M* = 0.98, *SD* = 0.03; poor readers: *M* = 0.97, *SD* = 0.05). These results indicate that, given the same amount of practice, poor readers and typical readers achieved similarly high accuracy across AO learning tasks.

### Decoding performance

#### English pseudoword decoding.

One participant was excluded from both the accuracy and latency analyses due to noncompliance with task instructions. Accuracy data were unavailable for one additional participant because of a video recording failure, and latency data were missing for another participant due to a data-saving error on the Pavlovia platform. Consequently, the final analyses were based on data from 32 participants.

The GLME model of decoding accuracy (Table 2) revealed a significant effect of group, with poor readers showing slightly lower accuracy (*M* = 0.95, *SD* = 0.10) than typical readers (*M* = 0.98, *SD* = 0.06), *β* = 4.78, *SE* = 2.21, *z* = 2.16, *p* = 0.03. Accuracy improved significantly across blocks for poor readers, while remaining stable for typical readers (Fig. 3A), as reflected in a significant group by block interaction, *β* = −0.15, *SE* = 0.04, *z* = −3.97, *p* < 0.001. There was also a main effect of block, *β* = 0.17, *SE* = 0.03, *z* = 6.67, *p* < 0.001. However, by the final block, accuracy between groups no longer differed significantly, *t*(22.04) = 0.36, *p* = 0.72.

For the analysis of decoding latencies, we used a simplified LME model because the model with a full random effects structure (slopes and intercepts) failed to converge due to low variance associated with the items factor. Consequently, the items variable was excluded from the final model reported in Table 2. The results yielded a significant effect of group: poor readers took longer to decode English pseudowords (*M* = 1.05 s, *SD* = 0.77 s) than typical readers (*M* = 0.47 s, *SD* = 0.22 s), *β* = −0.74, *SE* = 0.15, *t* = −4.84, *p* < 0.001 (Fig. 3B). A significant group by block interaction was also observed, *β* = 0.01, *SE* = 0.002, *t* = 6.41, *p* < 0.001, suggesting that the rate of improvement in decoding fluency differed by group. There was also a main effect of block, *β* = −0.05, *SE* = 0.001, *t* = −39.50, *p* < 0.001, indicating consistent fluency gains with repeated reading of the target pseudowords. However, poor readers remained significantly slower than typical readers in the final block, *t*(21.59) = 3.64, *p* = 0.001, suggesting persistent difficulties in achieving decoding fluency even after repeated practice.

#### AO word decoding.

**Figure 2:**
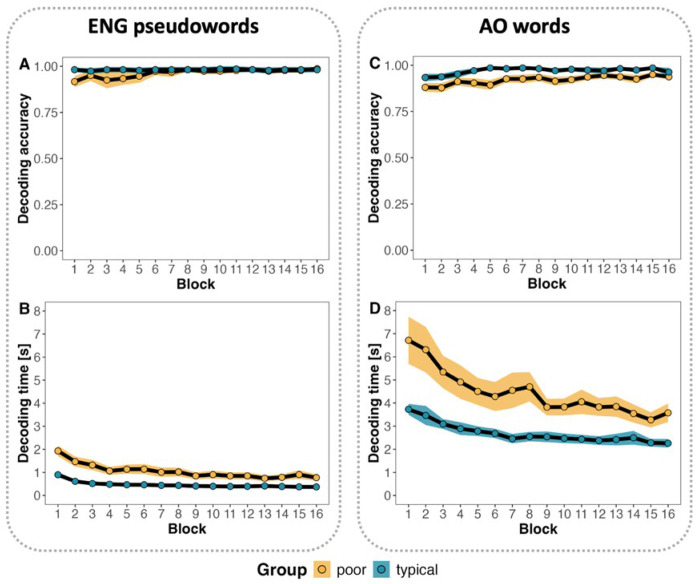
Decoding performance in the two research protocols, English pseudoword (A, B) and AO word (C, D) reading. The ribbon indicates the standard error around the mean.

Although overall accuracy of AO word decoding was high across groups (Fig. 2C), the GLME model with group and block as fixed effects and subjects and items as random effects revealed significant group differences (Table 3). Specifically, poor readers demonstrated lower decoding accuracy (*M* = 0.92, *SD* = 0.09) compared to typical readers (*M* = 0.97, *SD* = 0.06), *β* = 2.14, *SE* = 0.83, *z* = 2.58, *p* = 0.01. However, decoding accuracy improved significantly across blocks for both groups, *β* = 0.08, *SE* = 0.02, *z* = 4.66, *p* < 0.001. Notably, pairwise comparisons of accuracy in the final block showed no significant difference between groups, *t*(26.16) = 0.8, *p* = 0.41. No significant interaction was found between group and block.

An LME model of decoding times (Fig. 2D) also revealed a significant group effect: poor readers decoded more slowly (*M* = 4.48 s, *SD* = 2.66 s) than typical readers (*M* = 2.68 s, *SD* = 1.03 s), *β* = −0.46, *SE* = 0.12, *t* = −3.77, *p* < 0.001 (Table 3). A significant interaction between group and block was found as well, *β* = 0.004, *SE* = 0.002, *t* = 2.28, *p* = 0.022, suggesting group differences in the rate of improvement in decoding fluency. Additionally, a significant effect of block was observed, *β* = −0.03, *SE* = 0.001, *t* = −26.36, *p* < 0.001, indicating a gradual decrease in decoding times over the course of the task. Despite these gains, poor readers remained significantly slower than typical readers in the final block, *t*(25.67) = 3.33, *p* = 0.003, indicating persistent decoding challenges.

### Orthographic learning

#### English pseudoword learning outcomes.

##### Spelling.

**Figure 3:**
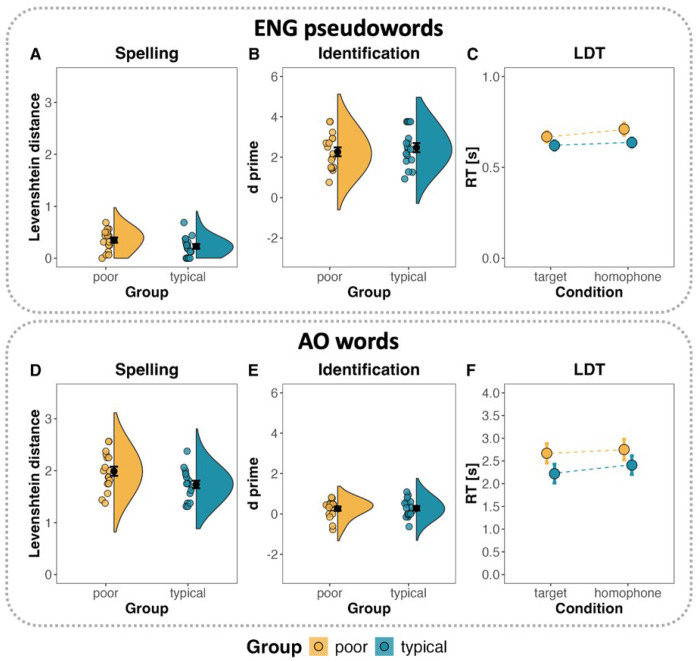
Orthographic learning performance across spelling, identification, and lexical decision tasks in the two research protocols, English pseudoword (A, B, C) and AO word (D, E, F) reading. The ribbon indicates the standard error around the mean.

An LME model with group as a fixed effect and random intercepts for subjects and items revealed no significant differences in Levenshtein distances (see Table 4). The average Levenshtein distance was small in both the poor reader group (*M* = 0.35, *SD* = 0.2) and the typical reader group (*M* = 0.23, *SD* = 0.18), indicating that participants’ spellings deviated by less than one letter from the target forms overall, reflecting good spelling accuracy (Fig. 3A).

##### Identification.

Overall d prime scores were high in both groups (poor readers: *M* = 2.27, *SD* = 0.89; typical readers: *M* = 2.48, *SD* = 0.91) (Fig. 3B). A Welch’s two-sample *t*-test revealed no significant difference between groups, *t*(31.98) = 0.67, *p* = 0.505, suggesting similarly strong ability across groups to discriminate target pseudowords from homophones.

##### LDT.

An LME model with random intercepts for subjects and items and a group by condition interaction on log-transformed LDT latency data revealed no significant effect of group, but a significant main effect of condition, *β* = 0.06, *SE* = 0.02, *t* = 3.15, *p* = 0.002 (Table 4). With both groups combined, participants responded faster to target pseudowords (*M* = 0.64, *SD* = 0.11) than to homophones (*M* = 0.67, *SD* = 0.13), with an average difference of 30 ms. This suggests that participants were sensitive to the learned orthographic forms (Fig. 3C).

#### AO word learning outcomes.

##### Spelling.

The full LME model with a maximal random-effects structure failed to converge. Consequently, we used a reduced model that included random intercepts for subjects and items (see Table 5). Results revealed significantly greater Levenshtein distances in the poor reader group (*M* = 1.99, *SD* = 0.36) than in the typical reader group (*M* = 1.73, *SD* = 0.29), *β* = −0.25, *SE* = 0.11, *t* = −2.25, *p* = 0.025. This indicates that the spellings produced by poor readers deviated more from the target forms (Fig. 3D).

##### Identification.

A Welch’s two-sample t-test on d prime scores revealed no significant group differences: *t*(31.99) = 0.14, *p* = 0.891. Poor readers (*M* = 0.26, *SD* = 0.44) and typical readers (*M* = 0.28, *SD* = 0.45) performed comparably, and overall d prime scores were low, suggesting weak memory representations for the target AO words (Fig. 3E).

##### LDT.

As with the spelling analysis, the full LME model failed to converge, so we used a reduced model with random intercepts for subjects and items, and included a group by condition interaction on log-transformed LDT latency data. The model revealed no significant interaction or main effects of group or condition (Table 5), indicating no strong orthographic learning of the AO words in either group (Fig. 3F).

##### Correlation analysis.

To examine whether phonological decoding skills were related to orthographic learning outcomes in the two research protocols (English pseudowords and AO words), we conducted Pearson correlation analyses using all dependent variables from each protocol: decoding accuracy, decoding latency, spelling task Levenshtein distances, identification task d prime scores, and the reaction time difference between homophones and targets in the LDT. In total, ten variables were included across the two protocols. Three sets of correlation analyses were carried out: 1) across all participants, 2) within the poor reader group only, and 3) within the typical reader group only. To control for multiple comparisons, *p* values were Bonferroni-corrected (α = 0.005; 0.05/10 dependent variables).

Contrary to predictions, phonological decoding measures from either protocol did not significantly correlate with orthographic learning outcomes in any of the analyses. The only significant effects were negative correlations between AO decoding accuracy and AO decoding latency, observed both in the typical reader group and across all participants (*r*(15) = −0.69, *p* = 0.002 and *r*(32) = −0.72, *p* < 0.001, respectively). Additionally, English pseudoword spelling performance was negatively correlated with English pseudoword identification d prime scores, again in the typical reader group and across all participants (*r*(15) = −0.84, *p* < 0.001 and *r*(32) = −0.53, *p* = 0.001, respectively). Finally, decoding latencies for English pseudowords and AO words were positively correlated when all participants were included (*r*(30) = 0.50, *p* = 0.004).

We further examined whether decoding and orthographic learning performance were associated with participants’ phonological and reading skills, as measured by standardized assessments (see Table 1). The only correlation to survive Bonferroni correction was a strong positive association between AO word decoding accuracy and performance on the CTOPP phoneme elision subtest, which measures the ability to manipulate phonological segments in spoken words. This effect was significant in the poor reader group (*r*(14) = 0.91, *p* < 0.001) and across all participants (*r*(31) = 0.80, *p* < 0.001). All other correlations between decoding and orthographic measures and standardized assessments were weak and nonsignificant.

##### Poor reader profiles.

**Figure 4:**
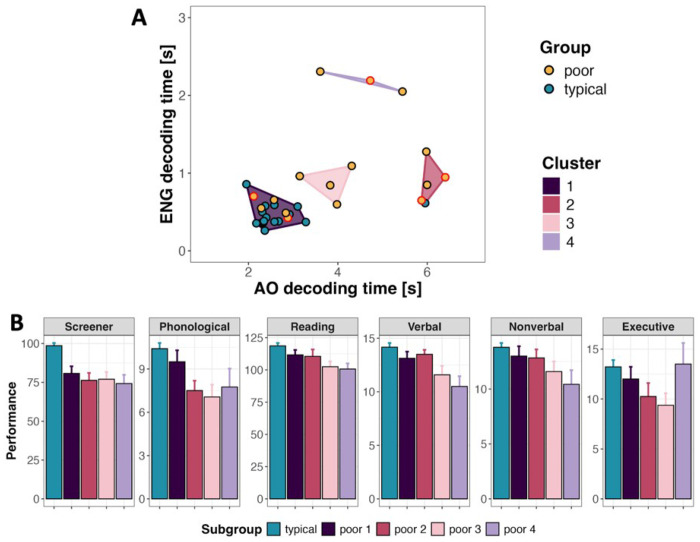
Participant clusters (A) and subgroup performance across cognitive assessment categories (B). Individuals with diagnosed dyslexia are indicated by red-outlined points.

Our analyses thus far indicate that the most reliable distinction between typical and poor readers lies in the decoding speed, both for English pseudowords and AO words, with these measures showing positive correlation. However, because correlational analyses do not capture population heterogeneity and poor readers are known to vary widely in etiology and symptom expression, we conducted an exploratory cluster analysis of decoding performance. Clusters were defined using English pseudoword and AO word decoding latencies and revealed four distinct profiles spanning both typical and poor readers, highlighting the limitations of binary group classifications (Fig. 4).

Cluster 1 included 16 of 17 typical readers and five poor readers (two with diagnosed dyslexia) and showed the fastest decoding in both tasks. Cluster 2 comprised one typical reader and four poor readers (two with diagnosed dyslexia) and was characterized by the slowest AO word decoding. Cluster 3 included four poor readers with intermediate AO decoding speed. Cluster 4 consisted of three poor readers (one with diagnosed dyslexia) who exhibited markedly slower English pseudoword decoding than all other groups.

To relate decoding heterogeneity to underlying cognitive abilities, composite scores were computed for each assessment domain (screening, phonological processing, text reading, verbal and nonverbal intelligence, and executive functioning (see Table 1). Qualitative comparisons contrasted poor readers in each cluster with the typical reader subgroup (cluster 1 plus one participant from cluster 2). Across clusters, poor readers performed below typical readers on screening measures, but cognitive profiles diverged. Cluster 1 poor readers (“fast English and AO decoders”) showed relatively strong phonological skills, potentially explaining their near-typical decoding latencies. Interestingly, this subgroup included two individuals with diagnosed dyslexia, perhaps representing cases of compensated dyslexia. Cluster 4 poor readers (“slow English decoders”) showed weaker verbal and nonverbal intelligence and text comprehension but relatively preserved executive functioning. In contrast, cluster 2 poor readers (“slow AO decoders”) demonstrated strong verbal and nonverbal intelligence and text comprehension but weaker executive control. Cluster 3 (“intermediate decoders”) showed intermediate performance across domains.

Comparison of orthographic learning outcomes across clusters showed minimal differences, except that cluster 4 poor readers (“slow English decoders”) were slower on the English LDT, and cluster 2 poor readers (“slow AO decoders”) were slower on the AO LDT, mirroring their decoding profiles.

Although exploratory, these results suggest that decoding performance reflects distinct constellations of cognitive strengths and weaknesses, and that readers’ diverse profiles can mask or compensate for deficits in different ways.

## Discussion

The present study examined whether adults with long-standing reading difficulties can successfully acquire new literacy skills, specifically in an unfamiliar orthography, and how their reading deficits influence phonological decoding and orthographic learning across both familiar (English pseudowords) and novel (AO) orthographies. Our results demonstrate that poor readers were able to learn novel grapheme–phoneme correspondences and achieve near-ceiling accuracy in decoding novel orthographic forms in both English and AO. Nevertheless, they remained consistently less fluent than typical readers in phonological decoding across both orthographies, even after repeated practice. Despite this persistent fluency gap, poor readers demonstrated orthographic learning outcomes comparable to those of matched typical readers in both protocols, although sensitivity to learned items, especially AO words, was poor in both groups. Correlation analyses provided little evidence for a direct link between decoding skills and orthographic learning. Notably, exploratory cluster analysis identified distinct subgroups of poor readers whose decoding profiles reflected different constellations of cognitive strengths and weaknesses. Taken together, these findings underscore the enduring challenges poor readers face in achieving decoding fluency in adulthood, while also demonstrating their capacity to acquire new orthographic systems with sufficient exposure.

### Phonological decoding in familiar and novel orthographies

Although poor readers eventually achieved decoding accuracy comparable to typical readers, they continued to lag in decoding fluency for both English pseudowords and AO words, even by the final block of practice. This result aligns with prior research showing that struggling readers face lifelong difficulties in phonological decoding, especially in speeded tasks (Elbro, Nielsen, & Petersen, 1994; Kitz & Tarver, 1989; Sabatini, 2002). A meta-analysis of adult reading studies similarly found that while accuracy deficits can occur, impairments are most pronounced in fluency measures (Reis et al., 2020). Comparable findings have also been reported in children, where accuracy may remain intact but fluency deficits persist, particularly in transparent orthographies (Suárez-Coalla et al., 2014).

These results indicate that adult poor readers’ primary limitation lies not in acquiring grapheme–phoneme correspondences or the alphabetic principle, but in executing decoding efficiently. The persistence of slowed decoding across familiar and novel orthographies points to a shared processing constraint rather than inadequate knowledge of English orthography or incomplete acquisition of AO.

One explanation rooted in the developmental literature attributes reduced fluency to underspecified orthographic representations, which hinder rapid word identification (Bailey et al., 2004; Castles & Nation, 2006; Ehri & Saltmarsh, 1995; Ehri, 2014; Suárez-Coalla et al., 2014). However, our orthographic learning results are inconsistent with this account: poor readers performed comparably to typical readers on tasks requiring recognition and recall of trained orthographic forms, suggesting similarly specified orthographic representations.

Instead, our findings align more closely with the phonological deficit hypothesis, which links reading impairments to weaknesses in phonological processing (Snowling, 1998; Wagner & Torgesen, 1987). This hypothesis, initially proposed to account for developmental dyslexia, is supported by extensive evidence showing that dyslexic adults score lower on phoneme awareness and phoneme manipulation tasks such as categorization, segmentation, and blending (Elbro, Nielsen, & Petersen, 1994; Pennington et al., 1990). Additional evidence links reading difficulties to reduced phonological short-term memory and lower verbal working memory spans (Abd Ghani & Gathercole, 2013; Pennington et al., 1990; Reiter, Tucha, & Lange, 2005; Smith-Spark et al., 2017), pointing to inefficiencies in how phonological information is accessed, retrieved, manipulated, and upkept in memory. Similarly, poor readers in our sample scored lower than typical readers on the CTOPP phoneme elision test (Table 1) measuring phonemic manipulation skills, which correlated positively with AO decoding performance. Moreover, exploratory cluster analysis revealed that poor readers with near-typical decoding fluency (including two individuals with diagnosed dyslexia) also exhibited stronger composite phonological skills.

Further insights come from comparing performance across different tasks: group differences were pronounced in the decoding task, which requires phonological and articulatory preparation, but minimal in the LDT, which can rely on orthographic activation alone. Poor readers were slower than typical readers by approximately 400 ms (English) and 1600 ms (AO) in the decoding task, compared to only 47 ms and 450 ms in the LDT, respectively, implicating phonological access and speech-related processes rather than orthographic representation as the primary source of slowed performance.

Related to the phonological deficit explanation is the rapid naming deficit hypothesis, which is based on observations that children with dyslexia often attain low scores on rapid automatized naming tasks, such as letter, digit, color, and object naming (Wolf & Bowers, 1999; Wolf, Bowers, & Biddle, 2000). According to this view, naming speed deficits arise from disruptions to precise timing mechanisms that coordinate phonological and visual processing. Naming speed has been shown to account for unique variance in reading performance beyond phonological skills and is more strongly associated with fluency than accuracy in word identification and letter–sound decoding (Manis, Doi, & Bhadha, 2000; Wolf, Bowers, & Biddle, 2000). Consistent with this account, our poor reader group scored lower on the CTOPP rapid letter naming test (Table 1), although this measure did not correlate with decoding or orthographic learning. Moreover, our study cannot determine whether reduced naming speed reflects a phonological impairment per se or a more general processing-speed limitation, as has also been reported in adults with reading difficulties (Sabatini, 2002).

In sum, our findings indicate that persistent fluency deficits of adult poor readers are best explained by underlying inefficiencies in phonological processing (potentially compounded by speed-related constraints), highlighting fluency as the central bottleneck in adult literacy acquisition.

### Orthographic learning in familiar and novel orthographies

The second research question asked whether lifelong reading difficulties affect adults’ ability to acquire novel orthographic forms in both familiar and unfamiliar writing systems. Across both groups, participants showed stronger learning in the English pseudoword protocol than in the AO protocol: Levenshtein distances in the spelling task, discriminability scores in the identification task, and reaction times in the lexical decision task were all better for English pseudowords than AO words. This advantage is likely explained by orthographic familiarity as well as stimulus differences. Specifically, English target pseudowords differed from their homophonous foils by only one grapheme (the vowel), whereas AO targets differed from foils by three consonant letters, making the AO tasks inherently more challenging. Crucially, however, no group differences emerged in orthographic learning outcomes for either orthography, with the sole exception of the AO spelling task, where poor readers produced more errors.

This pattern contrasts with the developmental literature, which consistently shows that children with reading difficulties struggle to acquire novel orthographic forms and often fail to establish durable orthographic representations (Bailey et al., 2004; Ehri & Saltmarsh, 1995; Share & Shalev, 2004; Suárez-Coalla et al., 2014), even when their phonological decoding skills are relatively intact (Castles & Coltheart, 1996; Hanley, Hastie, & Kay, 1992). Although research with adults is more limited, some studies have likewise shown that adults with dyslexia learn pseudowords more slowly than typical readers, particularly when spelling is irregular, and fail to encode and retrieve fine-grained orthographic detail in tasks such as picture identification, recognition, semantic categorization, and pseudoword rhyme (Howland & Liederman, 2013).

In contrast, our study did not find significant differences in orthographic learning outcomes between adult poor readers and matched typical readers in either familiar or novel orthography. This aligns with claims that poor readers may rely more on orthographic processing factors and visual memory than on phonological skills (Binder & Borecki, 2008; Kolinsky & Tossonian, 2023; Share, 1995), reflecting a general tendency to compensate for weak phonological skills. Indeed, when phonological coding demands are minimized, no differences are observed between children with and without dyslexia in tasks requiring visual recognition and recall of letters and words in an unfamiliar orthography (Hebrew) (Vellutino & Scanlon, 1982). Similarly, adults with dyslexia have been shown to achieve comparable outcomes for learning letter and symbol strings and to exhibit sensitivity to statistical properties such as chunk strength and positional frequencies in artificial grammar learning paradigms (Samara & Caravolas, 2017).

Taken together, our findings show that poor adult readers are capable of acquiring word-specific orthographic representations, particularly when phonological decoding is supported through repeated practice. This corroborates evidence that orthographic learning can occur through repeated exposures even when decoding is slow or effortful (Share, 2008) and highlights the potential for successful orthographic learning in adulthood, with implications for both adult literacy instruction and second language acquisition.

### Relationship between phonological decoding and orthographic learning

The third research question examined the relationship between decoding and orthographic learning outcomes. Contrary to predictions, the correlation analyses revealed no consistent association between measures of phonological decoding (accuracy or fluency) and orthographic learning performance. The only reliable relationship was observed between AO decoding accuracy and phoneme elision, underscoring the role of phonological awareness in supporting decoding accuracy.

These results suggest that phonological decoding and orthographic learning may rely on partially distinct mechanisms in adults. Whereas decoding depends heavily on phonological access, orthographic learning can proceed effectively once minimal accuracy thresholds are achieved, regardless of speed. This distinction complements theoretical accounts such as Share’s (1995) self-teaching hypothesis, which posits that phonological decoding serves as the gateway to orthographic learning. Thus, while our findings reaffirm the importance of decoding opportunities, they suggest that decoding efficiency itself may not directly constrain orthographic accuracy under supportive learning conditions where exposure is equated across groups.

### Heterogeneity among poor readers

Reading difficulties manifest in diverse ways, leading to highly heterogeneous profiles among poor readers (MacArthur et al., 2012; Mellard, Fall, & Mark, 2009; Morris et al., 1998). Our exploratory cluster analyses provide further insight into this variability. We identified four subgroups of poor readers who differed in decoding fluency: some showed slowed decoding primarily for AO words, others for English pseudowords, another performed comparably to typical readers across both measures, and a final group fell between these patterns. These subgroups also displayed distinct cognitive profiles. For instance, poor readers whose decoding latencies resembled those of typical readers showed relatively strong phonological skills. By contrast, those with slower decoding of English pseudowords demonstrated weaker general intelligence, while the subgroup with the longest AO decoding latencies showed reduced executive functioning. Taken together, these findings suggest that decoding performance may be shaped by compensatory mechanisms, with different constellations of cognitive strengths supporting reading in distinct ways. This heterogeneity underscores the limitations of treating poor readers as a uniform group and highlights the need for individualized approaches in both research and intervention.

### Limitations

This study has several limitations that provide important context for interpreting the findings and guiding future research. A first limitation concerns the use of an artificial orthography. This approach offers a high degree of experimental control over orthographic rules, exposure, and instruction—factors that are difficult to manage in naturalistic settings. However, learning to read in an AO is not equivalent to learning to read in a natural language. Thus, although the AO paradigm emulates certain aspects of early reading acquisition, the differences limit the extent to which direct comparisons can be drawn with child reading data. Future studies could complement AO paradigms with longitudinal designs tracking orthographic learning in natural languages, or hybrid approaches that combine experimental control with more ecologically valid reading contexts.

A second limitation relates to the heterogeneity of the poor reader sample. Reading difficulties manifest in diverse ways, and our data reflect this variability, with participants differing markedly in decoding performance. While we ensured that participants did not have comorbid conditions such as attention deficit disorders, the variability within our sample nonetheless underscores the need for caution in interpreting group-level results. Our exploratory cluster analysis highlighted meaningful subgroups of poor readers, but small sample sizes, in some cases as few as three individuals, constrained our ability to examine subgroup differences in orthographic learning more comprehensively. Larger-scale studies will be needed to validate these subgroups and to test whether distinct cognitive profiles predict different trajectories of orthographic learning.

## Conclusions

The present study demonstrates that adult poor readers retain persistent deficits in decoding fluency across both familiar and novel orthographies, even though they can acquire grapheme–phoneme correspondences and build accurate orthographic representations with practice. Decoding efficiency, while clearly impaired, does not appear to directly constrain orthographic learning outcomes under conditions of equal exposure. Importantly, the heterogeneity revealed by exploratory cluster analysis suggests that poor readers employ different mixtures of cognitive strengths and weaknesses to support decoding, pointing toward the value of more fine-grained profiling in both research and educational contexts.

## Supplementary Material

This is a list of supplementary files associated with this preprint. Click to download.

• Appendix.docx

## Figures and Tables

**Table 1:** Participant demographics, descriptive statistics (means and standard deviations in parentheses), and outcomes of between-group comparisons with the Welch’s t-test. Significant group differences after false discovery rate (FDR) correction are shown in **bold**.

Category	Measure	Typical	Poor	*t* value	*p* value
Demographics	Gender	12 F / 4 M / 1 Other	12 F / 3 M / 2 Other		
	Education level	3 some college / 6 Bachelor’s / 3 some graduate / 5 graduate degree	9 some college / 4 Bachelor’s / 1 some graduate / 3 graduate degree		
	Age (years)	25.12 (4.92)	28.41 (6.97)	1.59	0.122
Screener tasks	TOWRE Sight Word Efficiency (timed word reading)	105.77 (13.02)	83.3 (10.18)	5.61	**< 0.001**
	TOWRE Phonemic Decoding Efficiency (timed pseudoword reading)	107.36 (8.06)	80.95 (7.04)	10.18	**< 0.001**
	WRMT Word ID (untimed word reading)	103.71 (8.64)	88.95 (8.41)	5.05	**< 0.001**
	WRMT Word Attack (untimed pseudoword reading)	102.12 (10.98)	92.83 (9.82)	2.60	**0.014**
	ARHQ (%)	0.26 (0.06)	0.61 (0.13)	−10.84	**< 0.001**
Phonological processing	CTOPP phoneme elision	10.77 (1.04)	8.88 (2.1)	3.26	**0.004**
	CTOPP phoneme isolation	10.12 (2.35)	9.19 (2.2)	1.18	0.248
	CTOPP memory for digits	12.89 (3.04)	9.94 (4.08)	2.34	0.027
	CTOPP rapid letter naming	8.24 (3.44)	4.32 (2.34)	3.86	**0.001**
Reading comprehension	NDRT passage reading time	117 (18.03)	100.63 (16.62)	2.72	**0.011**
	NDRT passage comprehension accuracy	128.59 (11.94)	120.82 (11.41)	1.91	0.065
Verbal intelligence	WAIS verbal comprehension index	125.65 (15.15)	113.19 (11.05)	2.71	**0.011**
Nonverbal intelligence	WAIS perceptual organization index	125.35 (14.59)	111.69 (16.25)	2.54	**0.017**
Executive	DKEFS verbal fluency	15.12 (3.41)	12.50 (3.83)	2.07	0.047
	DKEFS inhibition	11.41 (3.18)	9.06 (3.30)	2.08	0.046

Note. TOWRE = Test of Word Reading Efficiency; WRMT = Woodcock Reading Mastery Test; ARHQ = Adult Reading History Questionnaire; CTOPP = Comprehensive Test of Phonological Processing; NDRT = Nelson-Denny Reading Test; WAIS = Wechsler Adult Intelligence Scale; DKEFS = Delis-Kaplan Executive Function System. Values represent standardized (TOWRE, WRMT, NDRT) or scaled (CTOPP, WAIS, DKEFS) scores.

**Table 2:** Results of the mixed-effects modeling of English pseudoword decoding performance (accuracy in log odds and log-transformed reading times). Significant effects are shown in **bold**.

Analysis type	Accuracy				Time			
Formula	glmer(acc ~ group*block + (1 + group | subject) + (1 | item))				lmer(rt ~ group*block + (1 + group | subject))			
Fixed Effects	*β*	*SE*	*z*	*p*	*β*	*SE*	*t*	*p*
(Intercept)	4.39	0.71	6.14	**< 0.001**	0.20	0.13	1.49	0.136
group	4.78	2.21	2.16	**0.031**	−0.74	0.15	−4.84	**< 0.001**
block	0.17	0.03	6.67	**< 0.001**	−0.05	0.001	−39.50	**< 0.001**
group x block	−0.15	0.04	−3.97	**< 0.001**	0.01	0.002	6.41	**< 0.001**
Random Effects								
σ^2^	3.29				0.11			
τ_00_ subject	4.07				0.29			
τ_00_ item	1.99							
τ_11_ subject x group	12.03				0.06			
N subject	32				32			
N item	16							
Observations	7996				7167			
Marginal R^2^ / Conditional R^2^	0.18 / 0.82				0.32 / 0.74			

**Table 3:** Results of the mixed-effects modeling of AO word decoding performance (accuracy in log odds and log-transformed reading times). Significant effects are shown in **bold**.

Analysis type	Accuracy				Time			
Formula	glmer(acc ~ group*block + (1 + group | subject) + (1 | item))				lmer(rt ~ group*block + (1 + group | subject) + (1 | item))			
Fixed Effects	*β*	*SE*	*z*	*p*	*β*	*SE*	*t*	*p*
(Intercept)	3.69	0.67	5.47	**< 0.001**	1.60	0.11	14.26	**< 0.001**
group	2.14	0.83	2.58	**0.010**	−0.46	0.12	−3.77	**< 0.001**
block	0.08	0.02	4.66	**< 0.001**	−0.03	0.001	−26.36	**< 0.001**
group x block	0.03	0.03	1.04	0.300	0.004	0.002	2.28	**0.022**
Random Effects								
σ^2^	3.29				0.10			
τ_00_ subject	4.36				0.19			
τ_00_ item	2.60				0.02			
τ_11_ subject x group	0.06				0.08			
N subject	34				34			
N item	16				16			
Observations	8454				7763			
Marginal R^2^ / Conditional R^2^	0.14 / 0.74				0.21 / 0.67			

**Table 4:** Results of the mixed-effects modeling of English pseudoword orthographic learning performance (spelling and LDT). Reaction times in seconds are log-transformed. Significant effects are shown in **bold**.

Analysis type	Spelling				LDT			
Formula	lmer(lev ~ group + (1 | subject) + (1 | item))				lmer(rt ~ group*condition + (1 | subject) + (1 | id))			
Fixed Effects	*β*	*SE*	*t*	*p*	*β*	*SE*	*t*	*p*
(Intercept)	0.35	0.06	5.52	**< 0.001**	−0.43	0.04	−10.19	**< 0.001**
group	−0.12	0.07	−1.84	0.067	−0.07	0.06	−1.17	0.244
condition					0.06	0.02	3.15	**0.002**
group x condition					−0.03	0.02	−1.49	0.138
Random Effects								
σ^2^	0.34				0.03			
τ_00_ subject	0.02				0.03			
τ_00_ item	0.03				0.00			
N subject	34				34			
N item	16				32			
Observations	544				993			
Marginal R^2^ / Conditional R^2^	0.01 / 0.12				0.04 / 0.55			

**Table 5:** Results of the mixed-effects modeling of AO word orthographic learning performance (spelling and LDT). Reaction times in seconds are log-transformed. Significant effects are shown in **bold**.

Analysis type	Spelling				LDT			
Formula	lmer(lev ~ group + (1 | subject) + (1 | item))				lmer(rt ~ group*condition + (1 | subject) + (1 | id))			
Fixed Effects	*β*	*SE*	*t*	*p*	*β*	*SE*	*t*	*p*
(Intercept)	1.99	0.11	17.51	**< 0.001**	0.86	0.09	9.61	**< 0.001**
group	−0.25	0.11	−2.25	**0.025**	−0.14	0.12	−1.13	0.259
condition					0.03	0.04	0.91	0.363
group x condition					0.05	0.04	1.27	0.204
Random Effects								
σ^2^	1.40				0.09			
τ_00_ subject	0.02				0.12			
τ_00_ item	0.11				0.004			
N subject	34				34			
N item	16				32			
Observations	544				998			
Marginal R^2^ / Conditional R^2^	0.01 / 0.09				0.02 / 0.60			

## Data Availability

All analyses were performed using in-house customized scripts in R and can be made available upon request.
